# Evaluation of Regional Vulnerability to Disasters by People of Ishikawa, Japan: A Cross Sectional Study Using National Health Insurance Data

**DOI:** 10.3390/ijerph15030507

**Published:** 2018-03-13

**Authors:** Makoto Fujiu, Yuma Morisaki, Junichi Takayama, Kiyoko Yanagihara, Tatsuya Nishino, Masahiko Sagae, Kohei Hirako

**Affiliations:** 1 Faculty of Environmental Design, Institute of Science and Engineering, Kanazawa University, Kanazawa 920-1192, Japan; takayama@staff.kanazawa-u.ac.jp (J.T.); tan378@se.kanazawa-u.ac.jp (T.N.); 2 Division of Environmental Design, Graduate School of Natural Science and Technology, Kanazawa University, Kanazawa 920-1192, Japan; yki20@stu.kanazawa-u.ac.jp; 3 Faculty of Health Sciences, Institute of Medical, Pharmaceutical and Health Sciences, Kanazawa University, Kanazawa 920-0942, Japan; kyana@mhs.mp.kanazawa-u.ac.jp; 4 Faculty of Economics and Management, Institute of Human and Social Sciences, Kanazawa University, Kanazawa 920-1192, Japan; sagae.masahiko@gmail.com; 5 Organization of Frontier Science and Innovation, Kanazawa University, Kanazawa 920-1192, Japan; hirako@staff.kanazawa-u.ac.jp

**Keywords:** KDB (national health insurance data), vulnerable people, earthquake, disaster evaluation, principal component analysis

## Abstract

The 2013 Partial Amendment of the Disaster Countermeasures Basic Law mandated that a roster of vulnerable persons during disasters be created, and further development of evacuation support is expected. In this study, the number of vulnerable people living in target analytical areas are identified in terms of neighborhood units by using the National Health Insurance Database to create a realistic and efficient evacuation support plan. Later, after considering the “vulnerability” of an area to earthquake disaster damage, a quantitative evaluation of the state of the disaster is performed using a principle component analysis that further divided the analytical target areas into neighborhood units to make a detailed determination of the number of disaster-vulnerable persons, the severity of the disaster, etc. The results of the disaster evaluation performed after considering the vulnerability of an area are that 628 disaster-vulnerable persons live in areas with a relatively higher disaster evaluation value.

## 1. Background and Purpose of the Study

### 1.1. Current State of Disaster-Vulnerable Persons

In the Great East Japan Earthquake that occurred on 11 March 2011, 60% of all deaths in the disaster areas were persons aged 60 and older, while 20% of all deaths in the disaster areas were persons with disabilities, showing that persons who required evacuation support were particularly severely injured [1]. In addition, according to the report, “Medical Treatment and Nursing in Disaster Areas—Current State and Issues after the Great East Japan Earthquake” [2], the handling of chronic diseases became an urgent challenge. In addition, according to that same report, dialysis treatment was unable to be provided in disaster areas owing to damaged medical facilities and water and power outages, so persons requiring artificial dialysis and persons with chronic diseases such as lifestyle-related diseases were harmed because they were unable to receive sufficient medical care. Considering these lesions from the Great East Japan Earthquake, a partial amendment was added to the Disaster Countermeasures Basic Law mandating that a roster be created for vulnerable persons requiring support for a series of actions during a disaster, such as evacuation to a safe location to protect themselves, and these persons included those who are elderly, disabled, injured/sick, nursing mothers, pregnant women, or foreigners [3]. Considering the situation, such as whether nursing or disability support is required, it was expected that creating a roster of disaster-vulnerable persons would allow information on those persons to be smoothly shared among aid workers and other relevant parties and could be put to great use for confirming safety and provide evacuation support. In addition to creating a roster of disaster-vulnerable persons, when envisioning an actual large-scale earthquake, it is critical to ascertain details on the devastation, such as the seismic intensity and disaster severity in an area, to provide evacuation support during a disaster more efficiently and effectively.

### 1.2. Purpose of This Study

This study focuses on and analyzes disaster-vulnerable persons who require rapid aid during an earthquake disaster. In addition, in light of the flow of disaster prevention plans in recent years, the area units were set very small to neighborhood units, and a detailed number of persons vulnerable to disaster, such as how many disaster-vulnerable persons exist in the neighborhood regions of the areas studied, is ascertained, and the number of citizens who could actually be injured in the disaster, including the severity of devastation in the neighborhood regions, were quantitatively analyzed. Analytical methods and procedures are as follows.

(i). The National Health Insurance Database, which contains a massive amount of medical data (hereinafter, “National health insurance data”), was used to determine the number of disaster-vulnerable persons in neighborhood units. In addition, from among the chronic illnesses described in the previous section as urgent challenges in the Great East Japan Earthquake, the diseases analyzed in this study were those requiring dialysis, such as ischemic heart disease, cerebrovascular disease and chronic renal disease.
(ii). With the units used to analyze the devastation severity set to neighborhood units, a quantitative analysis of the “external force” of the earthquake vibrations and the “vulnerability” of the neighborhood areas were quantitatively analyzed to evaluate the devastation. The “external force” of the earthquake vibrations was calculated using the value of the measured earthquake intensity from the earthquake vibration prediction map in the J-SHIS (Japan Seismic Hazard Information Station) earthquake hazard station that specified the earthquake source fault [4]. A neighborhood area’s “vulnerability” to earthquake damage was calculated using GIS and principal component analysis. The four surrogate variables during an earthquake disaster used when performing the principal component analysis were “population density (persons/km^2^)”, “elderly population density (persons/km^2^)”, “percentage of neighborhood area comprising building area”, and “the total length of roads in the neighborhood area (km)”.
(iii). From the results of (i), and (ii), it was possible to determine a detailed number of disaster-vulnerable persons living in the neighborhood region and the severity of the devastation by which they might be affected.

## 2. Previous Studies

### 2.1. Previous Studies on Disaster-Vulnerable Persons

There is one study discussing the evacuation support system for disaster-vulnerable persons. Because the death rate of elderly and disabled persons in the Great East Japan Earthquake was higher than that of healthy persons, this study discussed current issues and the format of future systems by performing an interview survey with respect to a Nankai Trough mega-quake to identify the current state of relief measures, such as the level of evacuation support provided to disaster-vulnerable persons in each region.

Nanba et al. [5] wrote the report, “Study related to Determination of the Present State of Disaster-Vulnerable Persons and Evacuation during Disasters—A Case Study Targeting the Central Region of East Hiroshima City”. This study identifies the evacuation locations in the central regions of East Hiroshima City in Hiroshima Prefecture and the areas where vulnerable persons are believed to be at risk, and analyzes the evacuation area. If a catastrophe occurs, the presence of danger around the place of life influences greatly the guarantee of safety in the case of no evacuation. On the other hand, the distance and the route to the refuge places determines safety in the case of evacuation. Especially, we must examine the evacuation place and evacuation route prudently for disaster vulnerable groups such as senior citizens. Grasp of the evacuation place and dangerous place necessary to consider because disaster vulnerable groups are increasing in aging society. The central area of Higashi-Hiroshima City is different from areas with the much country scenery such as the suburbs. Therefore, the distribution of population in both differs largely and population of the disaster vulnerable people, mainly senior citizens, differs depending upon the area. In this research, the refuge place and evacuation people were considered for the dangerous area in Higashi-Hiroshima City. Then, refuge places for disaster vulnerable groups such as senior citizens was considered.

Miki et al. wrote the report, “Identifying the Actual State of Tsunami Evacuation Behavior of Area Residents in the Great East Japan Earthquake: Analysis of Evacuees Including Disaster-Vulnerable Persons” [6]. Evacuation behavior characteristics in the Great East Japan Earthquake were identified from actual evacuation distances and routes, and, to contribute to future tsunami planning policies, the evacuation behavior characteristics of survivors during previous tsunamis were quantitatively determined from the results of a previous survey of those survivors.

Ito et al., wrote the report, “Analysis of the Present Physical and Mental State of Disaster-Vulnerable Persons in Model School Districts, Study of the Establishment and Capacity of Evacuation Facilities for Disaster-Vulnerable Persons in Cities A and B (Part 1)” [7]. Sugiyama et al. wrote the report, “Estimate of the Number of Disaster-Vulnerable Persons Corresponding to the Evacuation Rate by Evacuation Facility, Study of the Establishment and Capacity of Evacuation Facilities for Disaster-Vulnerable Persons in Cities A and B (Part 2)” [8]. Watanabe et al. wrote the report, “Analysis of the Capacity of Evacuation Facilities for Disaster-Vulnerable Persons, Study of the Establishment and Capacity of Evacuation Facilities for Disaster-Vulnerable Persons in Cities A and B (Part 3)” [9]. Ito et al., Sugiyama et al., and Watanabe et al. envisioned the actual circumstance during an earthquake to analyze the selection of evacuation facilities by physical and mental state and the evacuation location capacity by evacuation facility type in disaster-vulnerable persons in two cities, City A and City B, specifically, in disabled persons and persons certified to require nursing care.

### 2.2. Previous Studies of Disaster-Vulnerable Persons Using Massive Healthcare Data

While there are very few studies using National health insurance data to analyze disaster-vulnerable persons, there is a report by Tamamura et al. entitled, “Evacuation Simulation of Disaster-Vulnerable Persons during A Large-Scale Earthquake—Using the National Health Insurance Database”. National health insurance data from Komatsu City in Ishikawa Prefecture was used to perform a simulation of evacuation to the nearest nursing facilities and medical facilities by analyzing and determining the persons requiring nursing care, persons requiring high-cost medical care, and persons receiving artificial dialysis in Komatsu City [10]. There is also a report by Morisaki et al. entitled, “Discussion of Evacuation Facilities that Disaster-Vulnerable Persons with Serious Diseases Can Use in a Large-Scale Earthquake—Using the National Health Insurance Database of Hatoyama-machi [11]”. Using the J-SHIS earthquake hazard station, a complete or partial destruction rate curve, etc., the damage to buildings during a massive earthquake was ascertained, the available evacuation facilities were identified, and the actual state of disaster-vulnerable patients, particularly patients with serious diseases that complicate self-evacuation, was determined.

### 2.3. Previous Studies on Evaluating Devastation in Natural Disasters

There are hardly any previous reports that analyze the devastation of natural disasters, such as earthquake and landslides; however, there is a report by Ikenaga et al. that comprehensively analyzes natural disaster risks considering a reduced population in the future. This study considers the future population decline to comprehensively analyze the risk of disasters such as earthquake, flood, and landslide disasters and ascertains that risk in units of prefectures from the risk area population percentage and population reduction rate [12].

### 2.4. Actual State of Devastation Due to the East Japan Earthquake

The disaster prevention countermeasures that were proposed by Sobu et al. for patients with refractory diseases and based on the actual state of devastation in the East Japan Earthquake were studied in a survey of patients with refractory and chronic diseases in Iwate Prefecture to identify the state of devastation in detail. The problems encountered during a disaster were mentioned as “power outages”, “lack of water due to water outages”, “lack of gasoline”, and “no medicine”, and, specifically, there were those who stated that they could not receive dialysis owing to power and water outages or take their medication because of water outages [13].

Moreover, according to the Overall Condition and Political Issues of the Great East Japan Earthquake, there was such great damage during the East Japan earthquake that very few patients required surgery, but handling patients with refractory and chronic diseases was an urgent challenge [14]. In addition, because of the marked devastation to medical facilities, the disconnection of lifelines such as water and electricity, and the lack of pharmaceutical products due to confusion in distribution, DMAT (Disaster Medical Assistance Team) performed the transfer of patients within regions and over long distances.

### 2.5. Positioning of the Present Study Considering Previous Studies/Reports

The studies mentioned in Section 1 that focus on persons vulnerable in a disaster include those that discuss the format of future evacuation relief plans based on past large-scale earthquakes as well as those that identify the evacuation behavior of disaster-vulnerable persons and evacuation areas. In addition, while there are studies of the refugee capacity for housing disaster vulnerable persons, these studies do not consider realities such as the type of devastation disaster-vulnerable persons would be forced to face in an earthquake that could actually happen. Furthermore, of the studies of disaster-vulnerable persons, there are many that analyze persons requiring nursing care, but these studies do not focus on persons with the chronic diseases described as problematic in the introduction of this report. In addition, the study by Ikenaga et al. assesses the risk of natural disasters, but only in terms of prefectural units, without ascertaining that risk in the smaller area units of neighborhood units, which have been required for disaster prevention plans in recent years.

Considering the above, in the present study, the area units were set to neighborhood units, and the detailed number of persons vulnerable to disaster, such as how many disaster-vulnerable persons exist in the neighborhood regions of the areas studied, is ascertained from National health insurance data, and how many citizens could actually be injured in the disaster, including the severity of the devastation in the neighborhood regions, was quantitatively analyzed.

In addition, Tamamori and Morisaki et al. used National health insurance data to perform a study in disaster-vulnerable patients during a disaster where evacuation to medical facilities was simulated and usable medical facilities were identified; however, the present study uses the National health insurance data to assess devastation and is clearly different from the abovementioned study.

## 3. Analyzed Regions/Overview of Hakui City in Ishikawa Prefecture

**Figure 1:**
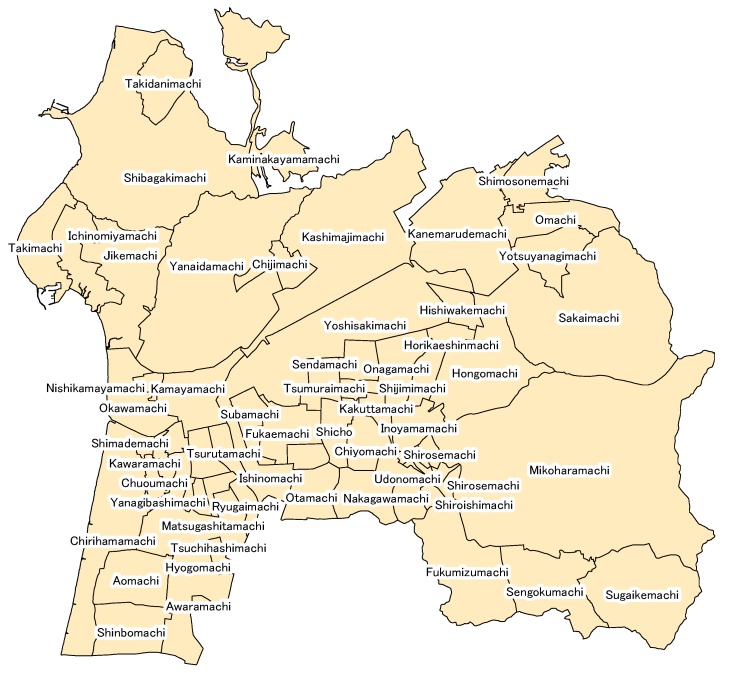
Hakui City in Ishikawa Prefecture and neighborhood segments.

The analyzed region in this study was Hakui City in Ishikawa Prefecture. Hakui City in Ishikawa Prefecture is located on the west side of the basal part of the Noto Peninsula. It borders Shikamachi, Hakuigun to the north; Himi City, Toyama Prefecture to the east; and Hodatsushimizu, Hakuigun to the south. It faces the Sea of Japan to the west, and Chirihama, the leading sight-seeing destination in the city, stretches from north to south. The population of Hakui City is 22,268 people (as of 1 April 2017), with 8530 households and an area of 81.85 km^2^. In addition, the elderly make up 36.2% of the population (as of 1 April 2017), a very high elderly rate [15]. Figure 1 shows Hakui City and its neighborhood segments. Hakui City in Ishikawa Prefecture can be divided into 65 neighborhood segments, and the subsequent analysis in the present study calculates the number of disaster-vulnerable persons and analyzes the devastation in the 65 segments shown in Figure 1.

## 4. Summary of National Health Insurance Data and Calculation of Disaster-Vulnerable Persons

This Section summarizes National health insurance data used for the calculation of disaster-vulnerable persons that is critical for assessing the devastation, establishes the diseases to be analyzed and shows the calculation results of the number of patients with those diseases.

### 4.1. Summary of National Health Insurance Data

**Figure 2:**
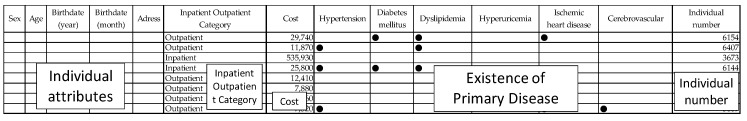
Example of MHLW form (Form 1-1) (source: created from the KDB data of Hakui City).

**Figure 3:**
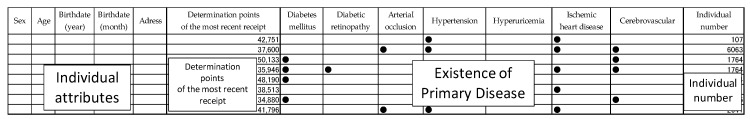
Example of MHLW form (Form 2-2) (source: created from the KDB data of Hakui City).

The present study uses National health insurance data, a massive set of healthcare data, for the calculation of disaster-vulnerable persons that is critical for assessing the devastation. “MHLW Form (Form 1-1)” and “MHLW Form (Form 2-2)”, in the ledger inside the National health insurance data, are used for the analysis. “MHLW Form (Form 1-1)” lists the medical fees used individually by month; it can also be used to find the name of the diseases diagnosed upon examination, the inpatient/outpatient category, and individual attributes. “MHLW Form (Form 2-2)” contains data picked up by patients requiring dialysis, and it individually lists the receipt determination score by month. Similar to MHLW Form (Form 1-1), Form 2-2 also lists the individual attributes, disease names diagnosed on examination, and individual attributes. Figure 2 shows an example of MHLW Form (Form 1-1), and Figure 3 shows an example of MHLW Form (Form 2-2). Note that, in Figure 1 and Figure 2, which show examples of the used National health insurance data, the “sex”, “age”, “birthdate (year)”, “birthdate (month)”, and “address” have been redacted because they could be used to identify the individual.

### 4.2. Establishment of the Diseases to Be Analyzed and Calculation Results

From “MHLW Form (Form 1-1)”, the diseases that can be calculated from National health insurance data are chronic diseases such as hypertension, diabetes mellitus, ischemic heart disease, and cerebrovascular diseases. In addition, diseases requiring dialysis, such as chronic renal disease, can be calculated from “MHLW Form (Form 2-2)”. As described in Section 1, because the handling of patients with chronic diseases was an urgent challenge during the Great East Japan Earthquake, the disaster-vulnerable persons addressed in the present study are patients with one of the three diseases that can be calculated from the National health insurance data, which are among the more urgent of the chronic diseases, and carry a higher risk of death: “patients with ischemic heart disease”, “patients with cerebrovascular disease”, and “patients requiring dialysis”. The analysis period was the full year FY 2015, subjects were at least 40 years old, and the sample size was 13,467 people.

The number of patients with ischemic heart disease, the number of patients with cerebrovascular disease, and the number of patients requiring dialysis were calculated for Hakui City based on National health insurance data, and these calculation results are shown in Table 1. From MHLW Form (Form 1-1), 1276 patients were diagnosed with ischemic heart disease, 1036 patients were diagnosed with cerebrovascular disease, and 56 patients were diagnosed as requiring dialysis. In addition, 1985 patients had at least one of the three diseases. Because a patient afflicted with at least one of the three abovementioned diseases was regarded as having sufficient urgency during a disaster, those 1985 patients were considered to be the “disaster-vulnerable patients” in the present study. The number of vulnerable patients by neighborhood is shown in the analysis results of Table 2.

The problem with National health insurance data in this study is that it only applies to persons who are enrolled in National Health Insurance. Therefore, the calculated number of patients might be an underestimation. Meanwhile, the reason that National health insurance data were used in this study is that they have the huge advantage of being very suitable for determining the number of patients by disease type in neighborhood units. It is difficult to use data other than National health insurance data to determine the number of patients in Hakui City in neighborhood units by injury or disease type, such as the number of patients with ischemic heart disease, the number of patients with cerebrovascular disease, and the number of patients requiring dialysis, so while there is the disadvantage that the number of disaster-vulnerable patients cannot be comprehensively determined in this study, the decision was made to use National health insurance data to comprehensively ascertain the number of disaster-vulnerable persons in Hakui City.

## 5. Devastation Assessment Considering Regional Vulnerability

In this section, the seismic intensity affecting Hakui City when the seismic center was identified is determined, and the vulnerability of each neighborhood region is quantitatively evaluated. Then, the devastation is assessed by considering the regional vulnerability in Hakui City. First, we describe the basic concepts when evaluating devastation. The Ochigata fault is specified in the present study since Hakui City in Ishikawa Prefecture is the target region, and the devastation is assessed from the “external force” of the earthquake vibrations and the “vulnerability” of the neighborhood regions of Hakui City to an earthquake disaster, which are variables describing the severity of the seismic intensity affecting each neighborhood of Hakui City. The following shows the relational expression between “devastation assessment”, “external force”, and “vulnerability”, which are used to assess the devastation in this study. In addition, the detailed analytical procedures and analysis results are shown in Section 5.1 and Section 5.2.

$$\begin{array}{l} D=H•\;V\left(Xi\right)\\ D:Devastation\;assessment\\ H:Hazard(External\text{​}force)\\ V(Xi):Vul\text{​}nerability(Vul\text{​}nerability(i)) \end{array}$$

### 5.1. Calculation of the External Force of Earthquake Vibrations

**Figure 4:**
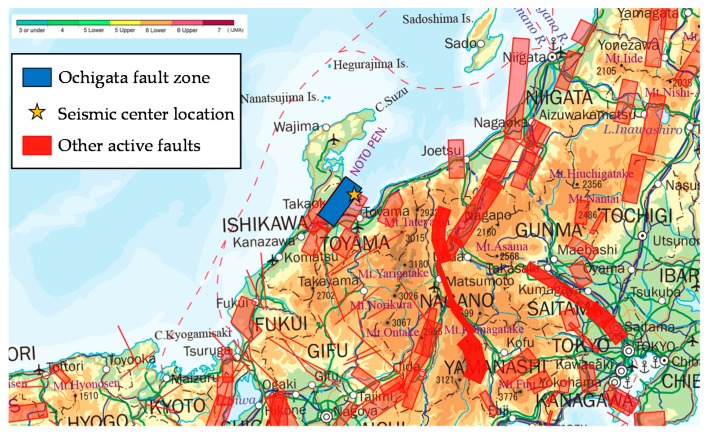
Ochigata fault zone and seismic center position.

**Figure 5:**
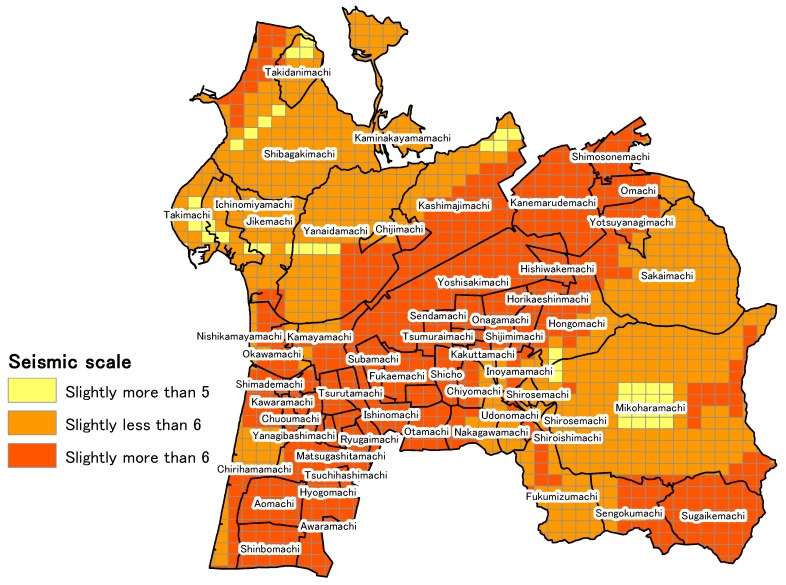
Visualizing the scale of the seismic intensity when an earthquake occurs in the Ochigata fault zone.

A J-SHIS earthquake hazard station is used to calculate the external force of earthquake vibrations in neighborhood units. The service provided by J-SHIS includes an earthquake vibration prediction map in which the earthquake source fault is specified, and it can specify the earthquake source fault envisioned when large seismic intensity is observed in Hakui City. In addition, the seismic intensity distribution when an earthquake occurs on that fault can also be determined. The faults from which large vibrations are anticipated to be observed in Hakui City are the Ochigata fault zone lying centrally in Ishikawa Prefecture and the Tonami Plain fault located on the northwest rim and southeast rim of the Tonami Plain. J-SHIS cannot predict earthquake vibrations when an earthquake occurs on two faults simultaneously, so the present study considers the situation where a large-scale earthquake occurs in the Ochigata fault zone, from which the largest vibrations are expected to be generated. Figure 4 shows the Ochigata fault zone and seismic center position. It is composed of the Kanto Hirano northwestern rim fault zone. GIS is used to visualize the severity of the earthquake intensity to affect Hakui City when an earthquake occurs at the seismic center of the Ochigata fault zone to grasp the severity of the vibrations in Hakui City. Figure 5 shows the results of the visualization. In Figure 5, vibration of slightly less than 6 to slightly more than 6 (on the Richter scale) affects the entire region of Hakui City. There are many locations in western and eastern Hakui City where an intensity of slightly less than 6 is observed, and vibrations of slightly greater than 6 are found to affect Asahimachi, where Hakui City Hall is located.

Next, the specific methods for calculating the external force of the earthquake vibration in neighborhood units is described. As can be seen in Figure 5, the information on the earthquake vibration obtained from J-SHIS is in 250 m mesh units, and the measured seismic intensity affecting each square of mesh can be determined. Since the “external force” of earthquake vibrations is handled as a surrogate marker for earthquake vibrations in each neighborhood region in the present study, the decision was made to calculate external force as the mean measured seismic intensity to affect each neighborhood region (the mean value of each measured seismic intensity in each mesh square in a neighborhood region). Details on the external force of the earthquake vibration by neighborhood are shown in the analysis results in Table 3.

### 5.2. Quantitative Evaluation of Earthquake “Vulnerability”

A principal component analysis, which is a multivariate analysis, was used to quantitatively evaluate earthquake vulnerability in neighborhood units. When measuring the vulnerabilities of a region to an earthquake disaster in this study, the three vulnerabilities considered were humans, buildings, and the roadway network, so the surrogate variables were “population density (persons/km^2^)”, “elderly population density (persons/km^2^)”, “percentage of neighborhood area comprising building area”, and “the total length of roads in the neighborhood area (km)”. The population density (persons/km^2^) and elderly population density (persons/km^2^) represent the vulnerabilities of a neighborhood region in terms of humans. In particular, the elderly population density is a surrogate variable that focuses on the elderly, who have a particularly high risk of being injured during a disaster. In addition, the percentage of neighborhood area comprising building area represents the vulnerability of a neighborhood region in terms of buildings, such as building collapse and structure fires. The total length of roads in the neighborhood area (km) is a surrogate variable established based on the concept that, when road length increases, the ease of evacuation improves, which reduces vulnerability.

A principal component analysis was performed with the abovementioned four variables as surrogate variables. The four variables were used as comprehensive markers, a quantitative analysis was performed, and the vulnerability by neighborhood was measured. Section 5.1 and Section 5.2 were calculated for each neighborhood using the results of the FY 2010 National Census [16]. In addition, the information on building area in Section 5.3 was obtained from the detailed map for 2014 from the Arc GIS Data Collection 2014, the information on roadway length in Section 5.4 was each obtained from the key statistics 2014 from the Arc GIS Data Collection 2014, and each variable was calculated from the geographical information analysis by GIS.

### 5.3. Quantitative Evaluation of Earthquake “Vulnerability” According to the Principal Component Analysis

**Figure 6:**
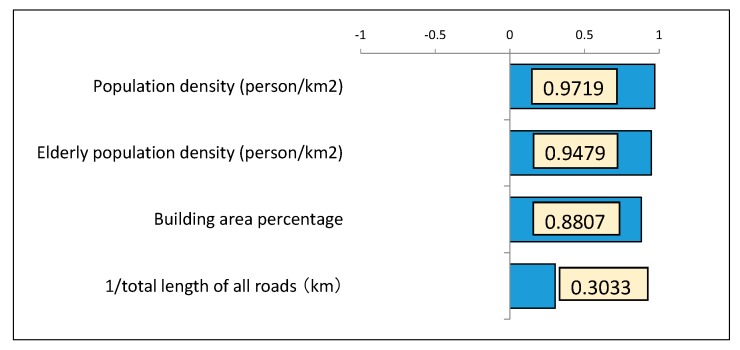
Principal component load of each variable in principal component 1.

Table 2 and Figure 6 show the results of the principal component analysis performed using each of the variables in Section 5.1, Section 5.2, Section 5.3 and Section 5.4 described in the previous section, that were obtained from the GIS geographical information analysis. Table 3 shows the eigenvalues, contribution ratio, and cumulative contribution ratio of principal components 1 to 4. In the present study, the decision is made to use the first principal component, showing a contribution ratio of 67.77%, as the vulnerability of each region. Figure 6 shows the principal component load of each variable in principal component 1. The load of the population density and elderly population density is extremely high, followed by the building area percentage, which is 0.8807. In addition, total roadway length had the lowest principal component load of 0.3033. The inverse of the variable of the total roadway length was taken to arrange the positive/negative orientation of the effect of each variable. The detailed measurement values for each neighborhood, such as the first primary component score, which is the comprehensive score indicator of vulnerability, and the value of each variable are all shown together in the analysis results in Table 2.

### 5.4. “Devastation Assessment” Calculated from External Force and Vulnerability

**Figure 7:**
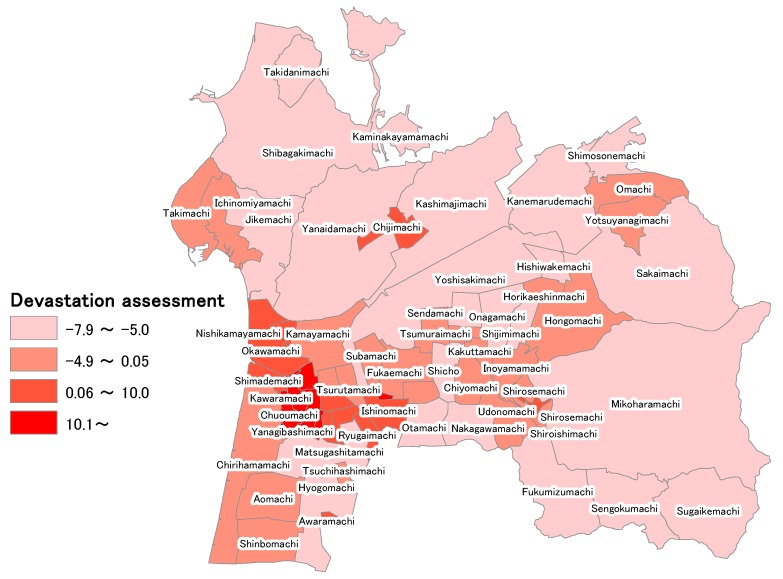
**Figure** **7.** Visualization of the devastation assessment in each neighborhood.

In Section 5.1, Section 5.2 and Section 5.3, the calculation of “external force” by the measured seismic intensity obtained from using J-SHIS, which was a calculation used as advance preparation for assessing devastation, and the quantitative assessment of “vulnerability” by principal component analysis considering regional characteristics of neighborhoods were performed. The devastation of each neighborhood considering regional vulnerability was assessed using the above results and Section 5.1. Table 2 shows the calculation results of the number of disaster-vulnerable persons by KDB (National health insurance data), the value of the “external force” calculated from the measured seismic intensity, the values of each variable when principal component analysis is performed, the score of the first principal component of the value component analysis, and the value of the disaster assessment. According to Table 2, the neighborhood with the highest number of disaster-vulnerable persons was Chirihama, with 197 people, and the neighborhood with the smallest number was Yanagibashimachi, with 0 people. In addition, the mean number of disaster-vulnerable persons per neighborhood was 30.54. In addition, according to Table 2, the region with the highest devastation assessment value was Wakakusamachi, at 43.06. Since the mean value was 0.06, Wakakusamachi was shown to be the neighborhood that would be most easily affected by earthquake damage. In addition, Wakakusamachi has 12 disaster-vulnerable persons, so 12 people living in Wakakusamachi would very easily be harmed in a disaster. The mean devastation assessment value was 0.06, and 19 of the 65 total neighborhoods exceeded the mean devastation assessment value. In addition, a total of 628 disaster-vulnerable persons live in the 19 regions that exceeded the mean devastation assessment value. Thus, of the 1985 disaster-vulnerable persons who were the subjects of this study, 31.64% were found to live in regions that exceeded the mean devastation assessment value. Next, GIS is used for visualizing the devastation assessment, focusing on its relationship with location in Hakui City. Figure 7 shows the visualization of the devastation assessment. The devastation can be visually assessed by performing the visualization in Figure 7. Neighborhoods that exceed the mean devastation assessment value of 0.06 were found to be concentrated in western Hakui City. In addition, because the devastation assessment was comparatively higher, and it was crowded in Chirihama, which had the highest number of disaster-vulnerable persons, and around Asahimachi, where the Hakui City Hall is located, it can be said that massive devastation might occur in highly populated regions of Hakui City when a large-scale earthquake occurs in the Ochigata fault zone.

**Figure 8:**
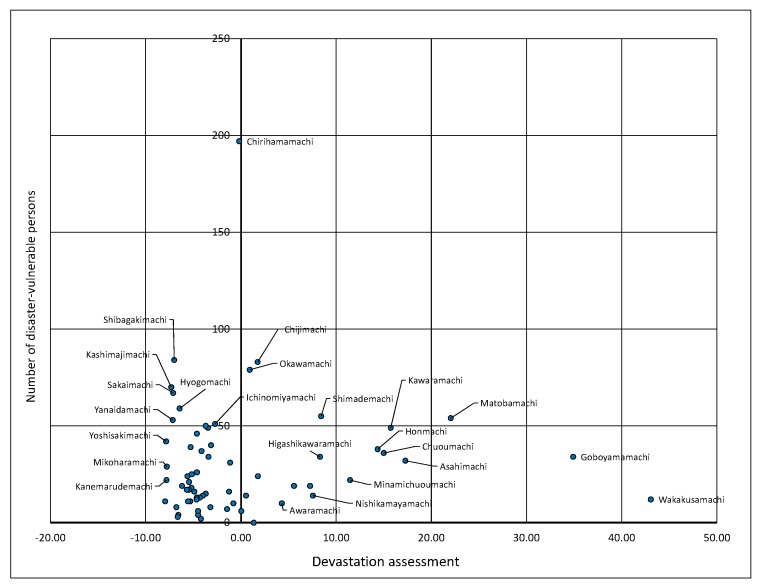
Relationship between the number of disaster-vulnerable persons and the devastation assessment.

In addition, the relationship between the number of disaster-vulnerable persons and devastation assessment value can be grasped visually. Figure 8 shows the relationship between the number of disaster-vulnerable persons and the devastation assessment in each neighborhood region. As can be seen in Figure 8, most points fall below 50 disaster-vulnerable persons and also below the mean devastation assessment value. In addition, the devastation assessment value is relatively high in the Shimademachi, Asahimachi, Kawaramachi, Matobamachi, and Chuomachi areas, and approximately 20–55 disaster-vulnerable persons live in each of these neighborhoods. Furthermore, the interpretation can be made that neighborhood regions with low devastation values tend to be concentrated in the northern and eastern parts of Hakui City. Although the devastation assessment is low around Omachi, Yotsuyanagimachi, Chijimachi, Takimachi, and Ichinomiyamachi, there are a few neighborhoods in which the devastation value is high in only one region when compared with the area around it.

## 6. Future Topics

As a future research topic, we will refine and develop the variables used in the principal component analysis. Specifically, an analysis will be performed with more variables using data from facilities such as factories, parks, and shelters. In this study, vulnerability is evaluated by principal component analysis, and authors think vulnerability can be expressed more precisely by using more variables. A sensitivity analysis is necessary to evaluate the parameters which most influence vulnerability.

## 7. Conclusions

This study focuses on and analyzes disaster-vulnerable persons who require rapid aid during an earthquake disaster, specifically, patients with ischemic heart disease, patients with cerebrovascular disease, and patients requiring dialysis. The number of patients were able to be determined in neighborhood units by using National health insurance data. In addition, using a geographical information analysis by GIS, a principal component analysis was used to perform a qualitative analysis of the vulnerability of neighborhoods. In addition, the surrogate marker of “external force” of the earthquake vibrations in neighborhood units was calculated using J-SHIS and measured seismic intensity. In addition to the abovementioned analyses, the devastation in Hakui City was assessed in neighborhood units, and the number of disaster-vulnerable persons was identified. The detailed devastation status related to disaster-vulnerable persons in this study, such as the number of disaster-vulnerable persons living in a neighborhood and the resulting degree of devastation, was determined. Furthermore, one possibility was discovered for evaluating “vulnerability” in neighborhood regions: a principal component analysis using the four variables of population density (persons/km^2^), elderly population density (persons/km^2^), percentage of neighborhood area comprising building area, and total length of roads in the neighborhood area(km). In addition, by using the devastation evaluation obtained through analysis with National health insurance data, the devastation assessment value was found to be relatively high in central areas of Hakui City, such as the Shimademachi, Asahimachi, Kawaramachi, Matobamachi, and Chuomachi areas. Approximately 20–55 disaster-vulnerable persons live in each of these neighborhoods. National health insurance data are actively used in the fields of medicine and health sciences, but the present study suggests the possibility of using National health insurance data in detailed disaster prevention plans in neighborhood units.

To reduce vulnerability, approaches from the viewpoint of town planning such as advancement of compact city and improvement of living space of the elderly are necessary. On the other hand, it is suggested that it is necessary to maintain appropriate infrastructure development level, even in a declining population society.

## Figures and Tables

**Table 1:** Calculation results of disaster-vulnerable patients using KDB data.

Target Disease	Number of Patients
Patients diagnosed with ischemic heart disease	1276
Patients diagnosed with cerebrovascular disease	1036
Patients diagnosed as requiring dialysis	56
Patients with at least 1 of these 3 diseases	1985

**Table 2:** Each analysis result and devastation assessment in each neighborhood, and the number of disaster-vulnerable persons.

Neighborhood Region	Population Density (persons/km^2^)	Elderly Population Density (persons/km^2^)	Building Area Percentage	1/Total Length of All Roads （km）	First Principal Component Score	Mean Measured Seismic Intensity	Devastation Assessment	Number of Disaster Vulnerable Persons
Wakakusamachi	4.35	3.53	4.21	−0.68	7.14	6.03	43.06	12
Goboyamamachi	4.03	3.53	2.61	−0.68	5.83	5.99	34.90	34
Matobamachi	1.88	3.19	1.46	−0.64	3.69	5.98	22.05	54
Asahimachi	1.62	1.98	1.31	−0.53	2.86	6.03	17.28	32
Kawaramachi	1.44	1.69	1.48	−0.57	2.61	6.04	15.74	49
Chuoumachi	0.96	1.16	2.40	−0.55	2.52	5.97	15.01	36
Honmachi	2.11	2.64	−0.79	−0.62	2.39	6.02	14.37	38
Minamichuoumachi	0.84	0.45	2.01	−0.46	1.93	5.96	11.47	22
Shimademachi	1.10	1.23	0.21	−0.51	1.42	5.94	8.43	55
Higashikawaramachi	0.97	0.92	0.59	−0.57	1.38	6.05	8.32	34
Nishikamayamachi	−0.04	−0.08	2.33	−0.41	1.28	5.91	7.57	14
Kamishirosemachi	0.17	0.12	0.01	0.90	1.25	5.81	7.28	19
Matsugashitamachi	0.79	0.41	0.36	−0.55	0.93	5.99	5.58	19
Awaramachi	0.11	0.36	0.08	−0.52	0.72	6.02	4.30	10
Ishinomachi	0.17	−0.27	0.85	−0.52	0.30	6.02	1.79	24
Chijimachi	0.19	0.37	0.16	−0.53	0.31	5.74	1.77	83
Yanagibashimachi	−0.35	−0.49	1.17	−0.35	0.23	6.01	1.36	0
Okawamachi	0.23	0.07	0.20	−0.44	0.16	5.88	0.94	79
Ryugaimachi	0.54	−0.27	−0.09	−0.53	0.09	5.99	0.55	14
Tsurutamachi	−0.29	−0.53	1.00	−0.48	0.01	6.01	0.05	6
Chirihamamachi	0.17	0.13	−0.12	−0.41	−0.03	5.88	−0.18	197
Tsuchihashimachi	−0.20	0.00	−0.08	−0.53	−0.13	6.01	−0.78	10
Inoyamamachi	−0.16	−0.09	−0.07	0.03	−0.19	5.83	−1.12	31
Horikaeshinmachi	−0.38	−0.29	0.22	−0.28	−0.20	6.23	−1.24	16
Higashimatobamachi	−0.10	−0.34	−0.03	−0.52	−0.24	6.03	−1.45	7
Ichinomiyamachi	−0.30	−0.31	−0.03	−0.36	−0.49	5.54	−2.71	51
Shinbomachi	−0.32	−0.37	−0.06	−0.19	−0.53	5.95	−3.13	40
Kakuttamachi	−0.54	−0.54	−0.47	0.44	−0.54	5.88	−3.19	8
Yotsuyanagimachi	−0.34	−0.36	−0.19	−0.02	−0.58	5.88	−3.39	34
Omachi	−0.34	−0.31	−0.17	−0.37	−0.58	5.93	−3.45	49
Takimachi	−0.32	−0.23	−0.43	−0.42	−0.68	5.44	−3.68	50
Shirosemachi	−0.53	−0.45	−0.42	0.34	−0.64	5.80	−3.69	15
Chiyomachi	−0.39	−0.45	−0.21	−0.32	−0.67	5.95	−3.98	14
Aomachi	−0.41	−0.46	−0.13	−0.28	−0.69	6.01	−4.13	37
Shiroishimachi	−0.56	−0.54	−0.73	−0.07	−0.71	5.88	−4.19	2
Tsumuraimachi	−0.35	−0.31	−0.47	−0.46	−0.72	6.00	−4.31	13
Uwaemachi	−0.40	−0.44	−0.54	−0.47	−0.74	6.04	−4.49	4
Mitsuyamachi	−0.42	−0.44	−0.60	−0.17	−0.75	6.02	−4.49	6
Kamayamachi	−0.45	−0.49	−0.21	−0.32	−0.77	5.95	−4.60	26
Hongomachi	−0.47	−0.35	−0.44	1.27	−0.78	5.92	−4.61	46
Udonomachi	−0.57	−0.51	−0.64	1.09	−0.80	5.76	−4.63	13
Subamachi	−0.30	−0.38	−0.53	−0.52	−0.78	5.97	−4.64	12
Fukaemachi	−0.42	−0.52	−0.33	−0.27	−0.82	5.97	−4.88	16
Shimosonemachi	−0.44	−0.44	−0.43	−0.45	−0.86	5.98	−5.14	25
Sendamachi	−0.46	−0.37	−0.56	−0.41	−0.85	6.11	−5.19	18
Jikemachi	−0.56	−0.53	−0.31	−0.27	−0.94	5.64	−5.27	39
Shijimimachi	−0.53	−0.51	−0.69	0.36	−0.85	6.21	−5.31	11
Shicho	−0.43	−0.41	−0.63	−0.40	−0.91	5.98	−5.44	21
Otamachi	−0.49	−0.48	−0.41	−0.49	−0.91	6.03	−5.46	17
Hishiwakemachi	−0.52	−0.45	−0.74	0.01	−0.89	6.20	−5.54	11
Onagamachi	−0.51	−0.48	−0.54	0.08	−0.91	6.15	−5.60	24
Takidanimachi	−0.58	−0.51	−0.57	−0.05	−1.01	5.59	−5.68	17
Nakagawamachi	−0.53	−0.49	−0.68	−0.29	−1.06	5.84	−6.18	19
Hyogomachi	−0.52	−0.53	−0.58	−0.44	−1.07	5.99	−6.43	59
Sengokumachi	−0.69	−0.65	−0.83	3.62	−1.11	5.91	−6.56	4
Kaminakayamamachi	−0.67	−0.63	−0.81	0.27	−1.16	5.70	−6.63	3
Fukumizumachi	−0.67	−0.62	−0.77	2.34	−1.18	5.75	−6.76	8
Shibagakimachi	−0.63	−0.59	−0.74	0.38	−1.25	5.62	−7.00	84
Sakaimachi	−0.64	−0.60	−0.78	1.99	−1.25	5.67	−7.10	67
Yanaidamachi	−0.63	−0.59	−0.71	−0.27	−1.25	5.71	−7.14	53
Kashimajimachi	−0.61	−0.56	−0.78	−0.10	−1.25	5.84	−7.31	70
Mikoharamachi	−0.69	−0.66	−0.88	5.02	−1.37	5.68	−7.78	29
Kanemarudemachi	−0.63	−0.59	−0.79	−0.33	−1.28	6.09	−7.81	22
Yoshisakimachi	−0.62	−0.61	−0.80	−0.21	−1.29	6.08	−7.84	42
Sugaikemachi	−0.69	−0.65	−0.89	1.64	−1.32	6.03	−7.94	11
						Average	0.06	30.54

**Table 3:** Eigenvalues, contribution ratio, and cumulative contribution ratio of principal components 1 to 4.

Primary Component	Eigen Value	Contribution Ration	Cumulative Contribution Ratio
1	2.711	67.77%	67.77%
2	0.941	23.53%	91.30%
3	0.317	7.92%	99.22%
4	0.031	0.78%	100.00%
